# Development and Validation of an HPLC Method for Simultaneous Assay of Potassium Guaiacolsulfonate and Sodium Benzoate in Pediatric Oral Powder

**DOI:** 10.1155/2019/6143061

**Published:** 2019-03-07

**Authors:** Thi Huong Hoa Le, Thi Hong Phung, Dinh Chi Le

**Affiliations:** ^1^National Institute of Drug Quality Control, Ministry of Health, Hanoi, Vietnam; ^2^Department of Analytical Chemistry and Toxicology, Hanoi University of Pharmacy, Hanoi, Vietnam

## Abstract

A novel HPLC method was developed and validated for simultaneous determination of potassium guaiacolsulfonate and sodium benzoate in pediatric oral powder. In this method, an analytical C8 column maintained at 25°C was used for chromatographic separation with a mixture of methanol and 0.02 M solution of tetrabutylammonium sulfate as the mobile phase. The composition of mobile phase was varied using a gradient program including an initial hold time of 7 minutes with methanol content maintained at 20% (v/v), followed by a linear gradient in 5.5 minutes in which methanol content was increased from 20% (v/v) to 50% (v/v) and a final hold time of 2.5 minutes with methanol content maintained at 20% (v/v). The total flow rate of mobile phase was maintained at 1.0 mL per minute. The UV detection was performed at 280 nm. Injection volume was set at 20 *µ*l. The method was fully validated in terms of specificity, linearity, precision, accuracy, and robustness according to requirements of current guidelines and was proved as reliable and suitable for the intended application.

## 1. Introduction

Guaiacolsulfonic acid, commonly used in the form of potassium guaiacolsulfonate, was a mixture of 1-hydroxy-2-methoxybenzene-4- and -5-sulfonic acid [1]. It is used as an expectorant for relieving symptoms of cough and mucus in the chest due to respiratory infections, asthma, colds, or hay fever. Guaiacolsulfonate works by thinning mucus (phlegm) in the lungs and making it less sticky and easier to cough up. This reduces chest congestion by making coughs more productive. Sodium benzoate is a preservative widely used in oral pharmaceutical preparation [2, 3] to inhibit the development of microorganism. Besides its antimicrobial property, sodium benzoate was also used to increase the solubility of active ingredients, like in the caffeine and sodium benzoate injection [4].

Potassium guaiacolsulfonate was determined in bulk active compounds by UV-Vis spectrometry [5] and by HPLC in an C18 column in both bulk active compounds [6] and in pharmaceutical dosage forms [7, 8]. Sodium benzoate was analyzed by volumetric titration [4] and by HPLC in the C18 column [2, 3, 9] in bulk material and dosage forms. Up to now, only one method for simultaneous analysis of potassium guaiacolsulfonate and sodium benzoate has been published using HPLC in the C18 column [8], however, the paper did not provide any information about the validation and performance capacity of this method. From the viewpoint of analytical chemistry in general, the AOAC International requires quantitative methods to meet certain minimal performance levels [10]. In pharmaceutical industry, supplying validation data of analytical methods to responsible authorities is now obligatory. Guidelines for analytical method validations were issued and available from several organizations, such as ICH [11] and FDA [12].

According to the guideline Q2 (R1) of ICH, “quantitative tests of the active moiety in samples of drug substance or drug product or other selected component(s) in the drug product” is one of the types of analytical procedures to be validated [11]. The validation of an analytical procedure ensures that the applied analytical technique, such as HPLC, shall give reliable and reproducible results. This process is very important because it provides information about the specificity, linearity, accuracy, precision, and sensitivity of the method, proving its suitability to the intended application.

In this study, an HPLC method using the C8 column was developed and validated for simultaneous assay of potassium guaiacolsulfonate and sodium benzoate in pediatric oral powder.

## 2. Materials and Methods

### 2.1. Instrumentation

The Shimadzu LC-20AT HPLC system (Shimadzu, Kyoto, Japan) was used for method development and validation. This system was equipped with a pump (model LC-20AD), a degasser (model DGU-20A5), a PDA detector (model SPD-M20A), an autosampler (model SIL-20ACHT), and a control module (model CBM-20 Alite). The chromatographic separation was executed on a Luna C8 column (250 × 4.6 mm, 5 *µ*m) of Phenomenex (Torrance, CA, USA). Software LCsolution Version 1.25 SP4 was used for data processing and evaluation.

### 2.2. Chemicals and Reagents

Pharmaceutical grade samples of potassium guaiacolsulfonate (purity 99.8%) and sodium benzoate (purity 98.6%) were kindly provided as a gift by Vietnam Pharmacochemistry Company (Hanoi, Vietnam). Mucibaby pediatric oral powder (containing 58.72 mg of potassium guaiacolsulfonate and 113.40 mg of sodium benzoate per sachet) was purchased from market. Methanol of HPLC grade, tetrabutylammonium sulfate of PA grade, and tetrabutylammonium hydrogen sulfate of PA grade were purchased form Merck Vietnam (Ho Chi Minh City, Vietnam).

### 2.3. Chromatographic Conditions

The mobile phase was a mixture of methanol and 0.002 M tetrabutylammonium sulfate solution whose composition was set by the gradient program in Table 1. The 0.002 M tetrabutylammonium sulfate solution was prepared by dissolving 1.16 g of tetrabutylammonium sulfate in 1000 mL of water, filtered through a 0.45 *µ*m membrane filter, and degassed by sonication for 15 minutes before used. The flow rate of the mobile phase was maintained at 1.0 mL/min. The analysis was carried out on a Shimadzu LC-20AT series HPLC system equipped with a PDA detector set at 280 nm for recording chromatograms. The chromatographic separation was conducted on a Luna C8 column (250 × 4.6 mm, 5 *µ*m) maintained at 25°C. The injection volume was 20 *µ*l.

### 2.4. Preparation of Standard Solution

Stock standard solutions of potassium guaiacolsulfonate (1.0 mg/mL) and sodium benzoate (2.0 mg/mL) were prepared by dissolving an accurately weighed quantity of corresponding reference standard in a mixture of methanol-water (20 : 80, v/v) as diluents. Working mix standard solutions were prepared by accurately diluting stock standard solutions to intended concentration with the same diluents. Standard solutions were filtered through a 0.45 *µ*m membrane filter before using for chromatographic analysis.

### 2.5. Preparation of Sample Solution

To analyze potassium guaiacolsulfonate and sodium benzoate in oral powder, 10 sachets were selected randomly, the average weight of powder per sachet was determined, and their contents were homogenized. An accurately weighed amount of homogenized powder equivalent to about 25 mg of potassium guaiacolsulfonate was dissolved and diluted to make 100 mL using a mixture of methanol-water (20 : 80, v/v) as the diluent. This solution was filtered through a 0.45 *µ*m membrane filter before using for chromatographic analysis.

### 2.6. Method Validation

#### 2.6.1. Specificity

Specificity is the ability of the analytical method to distinguish between the analyte(s) and the other components in the sample matrix [13]. In case of an HPLC method, it is assured by complete separation of peak(s) of analyte(s) from other peaks originated from the sample matrix. Specificity evaluation was done by injecting separately 20 *µ*l solution of standard, sample, placebo, and blank into the chromatographic system.

#### 2.6.2. Linearity

To evaluate the linearity of the method, mixed standard solutions of potassium guaiacolsulfonate and sodium benzoate were prepared by diluting stock standard solution with the mobile phase to obtain different exact concentrations of potassium guaiacolsulfonate (0.127, 0.204, 0.254, 0.305, and 0.382 mg/mL) and sodium benzoate (0.238, 0.381, 0.476, 0.571, and 0.714 mg/mL), corresponding to 50%, 80%, 100%, 120%, and 150% of target concentration, respectively. Three injections from each concentration were analyzed under the same conditions. Linear regression analysis was used to evaluate the linearity of the calibration curve by using the least square linear regression method, and the significance of linear regression was confirmed by one-way ANOVA test if *P*(Sig) < 0.05.

#### 2.6.3. Sensitivity

The limit of detection (LOD) and limit of quantitation (LOQ) of potassium guaiacolsulfonate and sodium benzoate were determined by analyzing different solutions of potassium guaiacolsulfonate and sodium benzoate and measuring the signal-to-noise ratio for each analyte. The limit of detection (LOD) is the concentration giving a signal-to-noise ratio of about 3 : 1, and the limit of quantitation (LOQ) is the concentration giving a signal-to-noise ratio of about 10 : 1, with RSD of less than 10% with triplicate analysis [14, 15].

#### 2.6.4. Accuracy

The accuracy of the method was determined by recovery studies for potassium guaiacolsulfonate and sodium benzoate from the placebo matrix [16]. Exact amounts of reference substances of potassium guaiacolsulfonate and sodium benzoate were mixed with the placebo matrix in such a way that the spiked samples, after preparation process, yielded solutions containing each analyte at three concentration levels, corresponding to 80%, 100%, and 120% of target concentration, i.e., about 0.200, 0.250, and 0.300 mg/mL with potassium guaiacolsulfonate and about 0.380, 0.47,5 and 0.570 mg/mL with sodium benzoate. At each concentration level, three samples were prepared and analyzed. The percentage recovery of added potassium guaiacolsulfonate and sodium benzoate and the RSD were calculated for each replicate samples.

#### 2.6.5. Precision

The proposed method was validated in terms of system precision and method precision according to current guidelines and published papers [11, 13, 17].

The system precision was determined by six measurements of mix standard solution containing each analyte at 100% of target concentration on the same day [13]. The method precision includes repeatability and intermediate precision [11]. They were determined by six measurements of sample solution containing each analyte at approximately 100% of target concentration on the same day and on two different days, respectively.

#### 2.6.6. Robustness

The robustness of the method was verified by investigating the effects caused by deliberate minor changes in experimental conditions to analyse results [18]. In this study, following changes were applied:

- Flow rate: ±0.2 mL/min
- Concentration of tetrabutylammonium sulfate solution: ±5%
- Gradient program: ±0.5 minute for initial hold time and final hold time, and gradient length of ±2% for initial and final percentages of organic solvent percentage in gradient slope

At each condition, a mix standard solution of potassium guaiacolsulfonate and sodium benzoate at 100% of target concentration and three sample solutions at approximately 100% target concentration were prepared and injected into the chromatography system. The robustness of the method was evaluated from the RSD of the peak area for each analyte after three consecutive injections of standard solution and the RSD of the content of potassium guaiacolsulfonate and sodium benzoate determined from sample solutions.

#### 2.6.7. Stability of Analytical Solution

The stability of analytical solutions was determined by analyzing the standard and sample preparations at 0 h, after one day in refrigerator, and at 25°C. For each solution, three injections were executed at each time, and the stability of analytical solutions was evaluated from the variation of the average peak area and RSD value of the peak area among repeated injections.

### 2.7. Data Processing

SPSS software (version 16.0) of IBM SPSS Software (IBM, Armonk, NY, USA) was used for statistical analysis of analytical results.

## 3. Results and Discussion

### 3.1. Method Development and Optimization

Certain information about physiochemical properties and chromatographic behaviors of potassium guaiacolsulfonate and sodium benzoate were obtained from literature research. The HPLC method was developed to select chromatographic conditions (stationary phase, mobile phase and wavelength for recording chromatogram of UV-Vis detector) and sample preparation procedure. For this purpose, preliminary trials were performed by varying the composition of mobile phase and optimizing chromatographic conditions on the Luna C8 Phenomenex column (250 × 4.6 mm, 5 *µ*m). The results of preliminary optimization were summarized in Table 2.

After the optimization, the chromatographic conditions as mentioned in Section 2.3 were used for the final method.

### 3.2. Method Validation

#### 3.2.1. Specificity

**Figure 1:**
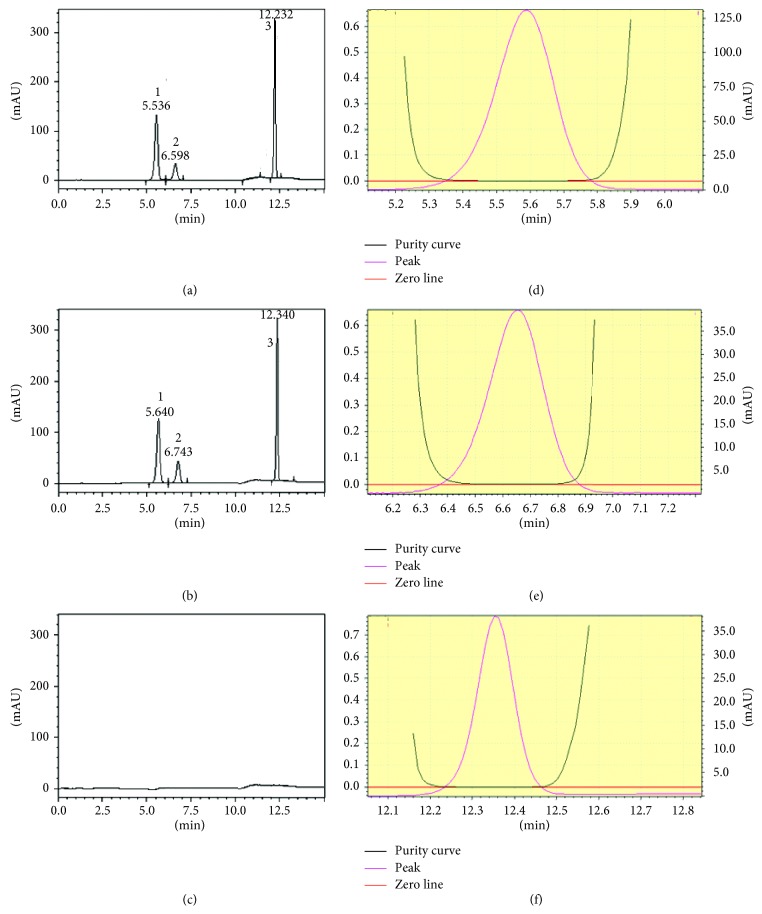
Chromatogram of mix standard solution (a), Mucibaby sample solution (b), placebo (c), and peak purity of analytes (peak 1 (d) and peak 2 (e) of potassium guaiacolsulfonate and peak of sodium benzoate (f)). Note: 1 and 2 denotespeak 1 and peak 2 of potassium guaiacolsulfonate, respectively. 3: Peak of sodium benzoate.

Specificity was evaluated by comparing the chromatograms of blank solution, placebo solution, standard solution, and sample solution (containing potassium guaiacolsulfonate and sodium benzoate at target concentration, i.e., 0.250 mg/mL and 0.475 mg/mL, respectively). For this purpose, 20 *μ*l from placebo solution, standard solution, and sample solution was injected into the HPLC system separately, and the chromatogram results are shown in Figures 1(a)–1(c). In selected chromatographic conditions, potassium guaiacolsulfonate was eluted into 2 well-separated peaks, and sodium benzoate was eluted in one peak. The two peaks of potassium guaiacolsulfonate were eluted before the peak of sodium benzoate. It can be observed from the peak purity analysis (Figures 1(d)–1(f)) that there are no coeluting peaks at the retention time of potassium guaiacolsulfonate and sodium benzoate to interfere with peaks of analytes. This result indicated that the peak of the analyte was pure, and this confirmed the specificity of the method.

#### 3.2.2. Linearity and Range

**Figure 2:**
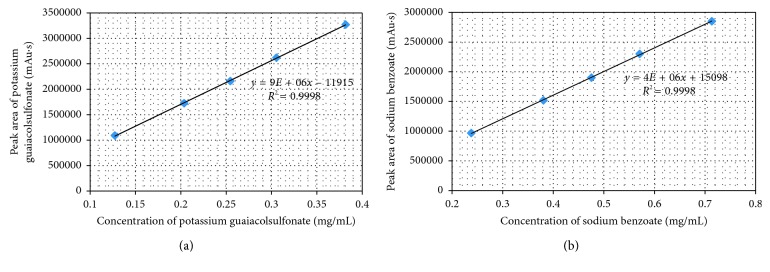
Calibration curves of potassium guaiacolsulfonate (a) and sodium benzoate (b).

Analytical method linearity is defined as the ability of the method to obtain test results that are directly proportional to the analyte concentration, within a specific range. The mean peak area obtained from the HPLC (in case of potassium guaiacolsulfonate, was the mean sum of areas of 2 peaks) was plotted against corresponding concentrations to obtain the calibration graph. The results of the linearity study (Figure 2) gave linear relationship over the concentration range of 0.127–0.382 mg/mL for potassium guaiacolsulfonate and of 0.238–0.714 mg/mL for sodium benzoate. From the regression analysis, the linear equation was obtained: *y* = 8587150*x* − 11915 for potassium guaiacolsulfonate and *y* = 3978487*x* + 15098 for sodium benzoate, and the coefficient of determination *R*^2^ was 0.999 for both analytes, indicating a linear relationship between the concentration of analyte and area under the peak. ANOVA analysis for both analytes (as shown in Tables 3 and 4) also proved that the regression model statistically significantly predicts the outcome variable (*P* < 0.05).

#### 3.2.3. Limit of Detection (LOD) and Limit of Quantification (LOQ)

The limit of detection (LOD) is the lowest amount of analyte in a sample that can be detected, but not necessarily quantitated, while the limit of quantification (LOQ) is the lowest amount of analyte in a sample that can be quantitatively determined with suitable precision [13]. For potassium guaiacolsulfonate, the concentration of injected solution at LOD and LOQ was 0.038 mg/mL and 0.127 mg/mL, equivalent to injected quantity of postassium guaiacolsulfonate of 0.76 *µ*g and 2.54 *µ*g, respectively. For sodium benzoate, the concentration of injected solution at LOD and LOQ was 0.071 mg/mL and 0.238 mg/mL, equivalent to injected quantity of sodium benzoate of 1.42 *µ*g and 4.76 *µ*g, respectively.

#### 3.2.4. Accuracy

The accuracy of an analytical method expresses the closeness of results obtained by that method to the true value. In this study, the results of recovery studies gave the recovery rate from 99.8% to 102.0% at all three levels for all the two analytes and RSD values at each level for each analyte varied from 0.1 to 0.5%, as shown in Table 5. These results were within the accepted limit for recovery (98.0% to 102.0%) and RSD (not more than 2.0%)

#### 3.2.5. Precision

The precision of the method is defined as “the closeness of agreement between a series of measurements obtained from multiple sampling of the same homogeneous sample under the prescribed conditions,” [14] and it is normally expressed as the relative standard deviation. In terms of system precision, the RSD of retention time, peak area, and performance of chromatographic system, represented by the number of theoretical plates and tailing factors, were all less than 2.0% and the number of theoretical plates was higher than 1000 for all analyte peaks, as shown in Tables 6 and 7. In terms of method precision, the RSD of assay results for potassium guaiacolsulfonate and sodium benzoate in evaluation of repeatability and intermediate precision were all less than 2.0%, as shown in Table 8. Therefore, the results of both system and method precision showed that the method is precise within the acceptable limits (not more than 2.0% for the RSD and the tailing factor and not less than 1000 for the number of theoretical plates).

#### 3.2.6. Robustness

The analytical method robustness was tested by evaluating the influence of minor modifications in HPLC conditions on system suitability parameters of the proposed method, as mentioned in Section 2.6.6.

The results of robustness testing, summarized in Table 9, showed that a minor change of method conditions, such as the composition of the mobile phase, flow rate, and gradient program, is robust within the acceptable limits (RSD less than 2.0%). In all modifications, good separation was achieved between the two components of potassium guaiacolsulfonate, as well as between them and sodium benzoate, and the RSD values of peak area obtained from repeated injections of standard solution and assay results for analytes obtained from sample solutions were all less than 2.0%. Furthermore, the minor changes applied in this robustness test produced no significant difference in content of potassium guaiacolsulfonate and sodium benzoate found in sample, as one-way ANOVA analysis found *F* < *F*_inscrit_ for both analytes (as shown in Table 10).

#### 3.2.7. Solution Stability

The percentage of recovery was within the range of 98.0% to 102.0%, and RSD was not more than 2.0%, indicating a good stability of the sample and standard solutions for 24  hr at both conditions, as shown in Table 11. These results proved that both analytes were stable in sample and standard solutions prepared as described in Sections 2.4 and 2.5 and the preparation procedure for sample and standard solution was suitable for intended application of the method.

## 4. Conclusion

In this study, a simple, accurate, precise, and robust HPLC method has been developed for simultaneous assay of potassium guaiacolsulfonate and sodium benzoate in pediatric oral powder. The method was validated according to the ICH guideline and proved to be suitable for the intended application, able to provide accurate and precise quantitative results under minor variation of chromatographic conditions.

## Figures and Tables

**Table 1:** Gradient program of mobile phase.

Time (minutes)	0.002 M solution of tetrabutylammonium sulfate (%)	Methanol (%)	Elution mode
0.0–7.0	80	20	Initial hold time
7.0–12.5	80 ⟶ 50	20 ⟶ 50	Linear gradient
12.5–15.0	80	20	Final hold time

**Table 2:** Results of preliminary optimization.

Column	Mobile phase	Elution mode	Flow rate	Observation	Result
Luna C8	Methanol-0.002 M solution of tetrabutylammonium sulfate (20 : 80, v/v)	Isocratic	1.0 mL/min	Elution force was too weak for sodium benzoate (retention time of sodium benzoate was more than 30 minutes)	Rejected

Luna C8	Acetonitrile-0.002 M solution of tetrabutylammonium sulfate (20 : 80, v/v)	Isocratic	1.0 mL/min	The two peaks of potassium guaiacolsulfonate were not completely resolute; retention time too long for sodium benzoate (more than 20 minutes)	Rejected

Luna C8	Methanol-0.002 M solution of tetrabutylammonium sulfate (50 : 50, v/v)	Isocratic	1.0 mL/min	Unable to distinguish the two components of potassium guaiacolsulfonate	Rejected

Luna C8	Methanol-0.005 M solution of tetrabutylammonium hydrogen sulfate (20 : 80, v/v)	Isocratic	1.0 mL/min	Poor and unstable peak shape for both analytes	Rejected

Luna C8	Methanol-0.002 M solution of tetrabutylammonium sulfate (20 : 80, v/v)	Gradient (the gradient program was presented in Table 1)	1.0 mL/min	Good resolution and peak shape for components of potassium guaiacolsulfonate; good resolution between potassium guaiacolsulfonate and sodium benzoate; acceptable analysis time (15 minutes)	Accepted

**Table 3:** Results of ANOVA analysis for calibration curve of potassium guaiacolsulfonate.

ANOVA^b^					
Model	Sum of squares	df	Mean square	*F*	Significance
1					
Regression	2.770*E*12	1	2.770*E*12	1.774*E*4	0.000^a^
Residual	4.685*E*8	3	1.562*E*8		
Total	2.770*E*12	4			

^a^Predictors: (constant), Conc. ^b^Dependent variable: area

**Table 4:** Results of ANOVA analysis for calibration curve of sodium benzoate.

ANOVA^b^					
Model	Sum of squares	df	Mean square	F	Significance
1					
Regression	2.077*E*12	1	2.077*E*12	1.552*E*4	0.000^a^
Residual	4.016*E*8	3	1.339*E*8		
Total	2.078*E*12	4			

^a^Predictors: (constant), Conc. ^b^Dependent variable: area

**Table 5:** Results of accuracy.

Spiked level (%)	Replicate number	Potassium guaiacolsulfonate				Sodium benzoate			
		Spiked amount of standard (mg)	Sum of peaks' area	Recovery (%)	Mean recovery, RSD (%)	Spiked amount of standard (mg)	Peak area	Recovery (%)	Mean recovery, RSD (%)
80	1	23.0	1988231	102.0	101.9	38.4	1532007	101.2	101.8
	2	20.6	1776475	101.7		38.6	1552884	102.0	
	3	20.5	1777035	101.9	0.1	38.6	1552844	102.0	0.5

100	1	25.3	2159862	100.7	100.3	48.0	1918840	101.4	101.7
	2	25.2	2199415	100.5		48.2	1934702	101.8	
	3	25.4	2199538	99.8	0.5	48.2	1935383	101.9	0.3

120	1	30.6	2648436	100.7	100.7	57.0	2260622	100.6	101.0
	2	30.9	2635143	100.6		57.2	2283777	101.3	
	3	30.8	2634916	100.9	0.2	57.3	2284080	101.1	0.4

**Table 6:** Results of system precision for potassium guaiacolsulfonate.

No. of injections	Retention time of potassium guaiacolsulfonate (minutes)		Peak area (mAu·s)		Asymmetry of peak		Number of theoretical plates		Resolution
	Peak 1	Peak 2	Peak 1	Peak 2	Peak 1	Peak 2	Peak 1	Peak 2	
1	5.640	6.743	1608422	587827	0.92	0.90	2035	2909	2.2
2	5.632	6.732	1607787	588263	0.91	0.91	2030	2900	2.2
3	5.626	6.727	1607305	588068	0.93	0.91	2025	2896	2.2
4	5.614	6.709	1608476	586795	0.93	0.89	2017	2880	2.2
5	5.594	6.682	1606855	586683	0.92	0.90	2002	2857	2.2
6	5.578	6.657	1605031	586820	0.93	0.91	1991	2836	2.2
Average	5.614	6.708	1607313	587409	0.92	0.90	2017	2880	2.2
RSD (%)	0.4	0.5	0.1	0.1	0.7	0.9	0.8	1.0	0.8

**Table 7:** Results of system precision for sodium benzoate.

No. of injection	Retention time of sodium benzoate (minutes)	Peak area (mAu·s)	Asymmetry of peak	Number of theoretical plates	Resolution
1	12.340	1905008	0.99	27071	14.0
2	12.386	1906997	0.99	27273	14.1
3	12.416	1906631	0.99	27405	14.2
4	12.344	1904799	0.98	27088	14.1
5	12.291	1905328	0.98	26856	14.0
6	12.334	1905038	0.99	27044	14.2
Average	12.352	1905634	0.99	27123	14.1
RSD (%)	0.4	0.1	0.5	0.7	0.6

**Table 8:** Results of repeatability and intermediate precision.

No. of sample solution	Sample weight (g)	Content of potassium guaiacolsulfonate in oral powder (%, comparing to labeled amount)	Content of sodium benzoate in oral powder (%, comparing to labeled amount)
*Day 1, analyst 1*			
1	0.6152	98.1	101.8
2	0.6224	100.8	100.0
3	0.6369	99.6	101.1
4	0.6102	98.7	102.7
5	0.6224	100.7	99.9
6	0.6394	99.4	100.8
Average (1–6)		99.6	101.0
RSD (%) (1–6)		1.1	1.1

*Day 2, analyst 2*			
7	0.6542	100.3	101.9
8	0.6395	99.2	101.5
9	0.6452	101.3	101.3
10	0.6531	100.4	102.3
11	0.6407	99.0	101.3
12	0.6446	101.6	99.4
Average (1–12)		99.9	101.2
RSD (%) (1–12)		1.1	1.0

Note: results obtained in day 1 by analyst 1 (sample no. 1–6) were used for evaluating repeatability, and those obtained in day 1 and day 2 (sample no. 1–12) were used together for evaluating intermediate precision.

**Table 9:** Results of robustness.

Variation	Specific condition	Potassium guaiacolsulfonate			Sodium benzoate	
		RSD (%) for area of peak 1	RSD (%) for area of peak 2	RSD (%) for content in oral powder	RSD (%) for peak area	RSD (%) for content in oral powder
Flow rate (mL/min)	0.8	0.12	0.01	0.09	0.64	0.98
	1.0 (normal)	0.03	0.04	0.06	1.36	0.90
	1.2	0.13	0.23	0.05	0.72	0.88
Concentration of tetrabutyl-ammonium- sulfate solution	0.0019 M	0.03	0.04	0.06	1.36	0.90
	0.0020 M (normal)	0.07	0.18	0.05	0.46	0.84
	0.0021 M	0.02	0.15	0.11	0.60	0.56

*Gradient program*						
Initial hold time (min)	6.5	0.04	0.21	0.02	0.47	0.45
	7.0 (normal)	0.02	0.15	0.01	0.35	0.64
	7.5	0.03	0.04	0.06	1.36	0.90
Gradient length (min)	5.0	0.03	0.11	0.02	0.40	0.56
	5.5 (normal)	0.03	0.17	0.05	0.86	0.77
	6.0	0.05	0.08	0.03	0.38	0.81
Final hold time (min)	2.0	0.03	0.04	0.06	1.36	0.90
	2.5 (normal)	0.03	0.12	0.04	1.26	0.65
	3.0	0.06	0.15	0.04	0.98	1.05
Initial percentage of methanol in gradient slope	18%	0.03	0.04	0.06	1.36	0.90
	20% (normal)	0.04	0.07	0.02	1.09	0.94
	22%	0.03	0.16	0.02	0.76	0.58
Final percentage of methanol in gradient slope	48%	0.03	0.04	0.06	1.36	0.90
	50% (normal)	0.04	0.08	0.02	0.61	0.61
	52%	0.12	0.01	0.09	0.64	0.98

**Table 10:** Results of ANOVA analysis for content of potassium guaiacolsulfonate and sodium benzoate.

ANOVA					
	Sum of squares	df	Mean square	*F*	Sig.
Guaiacol					
Between groups	4,603	20	0.230	0.322	0.996
Within groups	30,000	42	0.714		
Total	34,603	62			

Benzoate					
Between groups	7,556	20	0.378	0.881	0.609
Within groups	18,000	42	0.429		
Total	25,556	62			

**Table 11:** Results of stability studies.

Studies	Average retention time (minutes)		Average peak area (mAu·s)		Average asymmetry of peak		Average number of theoretical plate		RSD (%) of peak area		Recovery (%)	
	Peak 1	Peak 2	Peak 1	Peak 2	Peak 1	Peak 2	Peak 1	Peak 2	Peak 1	Peak 2	Peak 1	Peak 2
*Potassium guaiacolsulfonate*												
Standard solution												
0 h	5.633	6.734	1607838	588052	0.92	0.91	2030	2902	0.03	0.04	—	—
24 h at refrigerator	5.636	6.734	1607531	587210	0.92	0.90	2032	2902	0.03	0.05	100.0	99.9
24 h at 25°C	5.629	6.731	1606491	585247	0.92	0.91	2028	2899	0.02	0.02	99.9	99.5
Sample solution												
0 h	5.630	6.736	1605436	585067	0.93	0.90	2028	2903	0.01	0.01	—	—
24 h at refrigerator	5.631	6.730	1604722	584850	0.93	0.91	2029	2898	0.02	0.01	100.0	100.0
24 h at 25°C	5.629	7.735	1603874	583290	0.92	0.91	2027	2902	0.05	0.05	99.9	99.7

*Sodium benzoate*												
Standard solution												
0 h	12.380		1906212		0.99		27250		0.06		—	
24 h at refrigerator	12.372		1905065		0.98		27214		0.02		99.9	
24 h at 25°C	12.366		1903771		0.99		27186		0.01		99.9	
Sample solution												
0 h	12.375		1904980		0.99		27226		0.01		—	
24 h at refrigerator	12.381		1904544		0.99		27249		0.02		100.0	
24 h at 25°C	12.367		1903276		0.99		27192		0.01		99.9	

## Data Availability

The data used to support the findings of this study are available from the corresponding author upon request.
